# In-Vitro and In-Vivo Establishment and Characterization of Bioluminescent Orthotopic Chemotherapy-Resistant Human Osteosarcoma Models in NSG Mice

**DOI:** 10.3390/cancers11070997

**Published:** 2019-07-17

**Authors:** Maria Eugénia Marques da Costa, Antonin Marchais, Anne Gomez-Brouchet, Bastien Job, Noémie Assoun, Estelle Daudigeos-Dubus, Olivia Fromigué, Conceição Santos, Birgit Geoerger, Nathalie Gaspar

**Affiliations:** 1 National Centre for Scientific Research (CNRS), UMR8203, Gustave Roussy, 94805 Villejuif, France; 2 University of Paris-Saclay, 91190 Saint-Aubin, France; 3 University of Paris Sud, 91400 Orsay, France; 4 Department of Biology, Centre for Environmental and Marine Studies (CESAM), University of Aveiro, 3810 Aveiro, Portugal; 5 IUCT-Oncopole, CHU and University of Toulouse, Pathology department, 31100 Toulouse, France; 6 National Centre for Scientific Research (CNRS), UMR5089, 31077 Toulouse, France; 7 National Institute for Health and Medical Research (INSERM), US23, Gustave Roussy, 94805 Villejuif, France; 8 National Institute for Health and Medical Research (INSERM), UMR981, Gustave Roussy, 94805 Villejuif, France; 9 Department of Biology, Faculty of Sciences, University of Porto, 4000 Porto, Portugal; 10 Department of Oncology for Child and Adolescent, Gustave Roussy, 94805 Villejuif, France

**Keywords:** bone tumor, cell-derived xenograft, bioluminescence, resistance, methotrexate, doxorubicin, MDR1, DHFR

## Abstract

Osteosarcoma, the most common bone malignancy with a peak incidence at adolescence, had no survival improvement since decades. Persistent problems are chemo-resistance and metastatic spread. We developed in-vitro osteosarcoma models resistant to chemotherapy and in-vivo bioluminescent orthotopic cell-derived-xenografts (CDX). Continuous increasing drug concentration cultures in-vitro resulted in five methotrexate (MTX)-resistant and one doxorubicin (DOXO)-resistant cell lines. Resistance persisted after drug removal except for MG-63. Different resistance mechanisms were identified, affecting drug transport and action mechanisms specific to methotrexate (RFC/SCL19A1 decrease, DHFR up-regulation) for MTX-resistant lines, or a multi-drug phenomenon (PgP up-regulation) for HOS-R/DOXO. Differential analysis of copy number abnormalities (aCGH) and gene expression (RNAseq) revealed changes of several chromosomic regions translated at transcriptomic level depending on drug and cell line, as well as different pathways implicated in invasive and metastatic potential (e.g., Fas, Metalloproteinases) and immunity (enrichment in HLA cluster genes in 6p21.3) in HOS-R/DOXO. Resistant-CDX models (HOS-R/MTX, HOS-R/DOXO and Saos-2-B-R/MTX) injected intratibially into NSG mice behaved as their parental counterpart at primary tumor site; however, they exhibited a slower growth rate and lower metastatic spread, although they retained resistance and CGH main characteristics without drug pressure. These models represent valuable tools to explore resistance mechanisms and new therapies in osteosarcoma.

## 1. Introduction

Osteosarcoma is the first primary malignant bone tumor that predominantly occurs during adolescence [1,2]. Standard treatment combines neoadjuvant and post-operative chemotherapy with complete surgery of all primary and metastatic sites. No improvement has been seen in prognosis for almost five decades. Treatment failure is usually due to metastatic relapse. The presence of metastases at diagnosis and poor histological response to neoadjuvant chemotherapy are risk factors of relapse [1,3,4,5]. Resistance to therapy, both intrinsic (phenomenon present prior to chemotherapy administration) and acquired (revealed after chemotherapy administration) contributes to treatment failure and recurrence. Several mechanisms of chemo-resistance have been reported in osteosarcoma, from drug specific mechanisms to broader mechanisms [6]. However, the link between resistance to chemotherapy and metastatic phenotype remains unclear.

The aim of the study was to establish and characterize osteosarcoma models resistant to common chemotherapeutic agents and analyze their behavior comparatively to their parental counterparts in-vitro and in-vivo in a bone orthotopic setting.

## 2. Results

### 2.1. Development of In-Vitro Osteosarcoma Cell Lines Resistant to Chemotherapy

**Figure 1:**
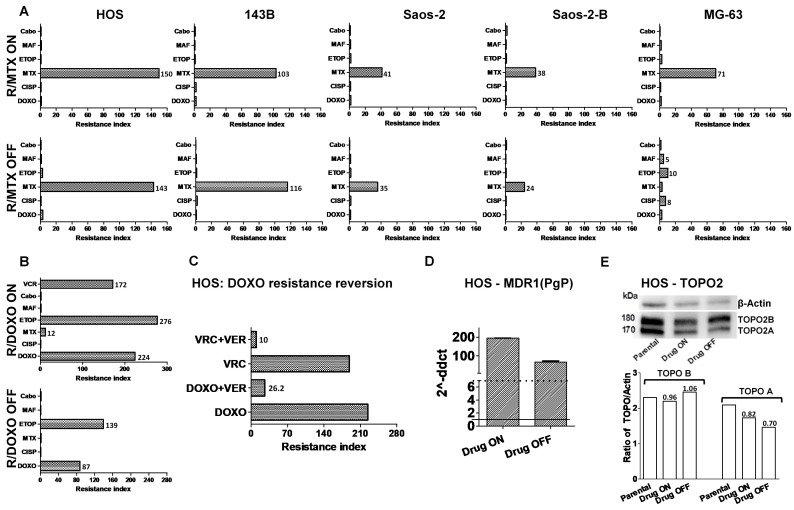
Acquired in-vitro resistance to methotrexate (MTX) and doxorubicin (DOXO) in osteosarcoma cell lines and cross-resistance with other chemotherapeutic agents used in osteosarcoma. Resistance Index (RI) is defined as the ratio IC50 of resistant line/IC50 of the corresponding parental line. IC50 - Half-maximal inhibitory concentration. (**A**) RI of MTX and cross-resistance to other drugs used in osteosarcoma (doxorubicin, methotrexate, etoposide, cisplatin, mafosfamide, and cabozantinib) for the MTX resistant lines (R/MTX)—Drug-ON and Drug OFF (at 9 weeks); (**B**) RI of DOXO and cross-resistance to other drugs used in osteosarcoma (doxorubicin, methotrexate, etoposide, cisplatin, mafosfamide, cabozantinib, and vincristine) for the DOXO resistant lines (HOS-R/DOXO)— Drug-ON and Drug OFF (at nine weeks). Vincristine was also used to treat the resistant line Drug-ON; (**C**) resistance reversion by P-glycoprotein (PgP) inhibitors in HOS-R/DOXO with the PgP inhibitor Verapamil (VER); (**D**) Multidrug resistance polypeptide 1 (MDR1/ABCB1 or PgP) mRNA expression in HOS-R/DOXO Drug-ON and Drug-OFF by RT-qPCR (reverse transcription– quantitative polymerase chain reaction) compared to the parental line equal to expression level of 1; (**E**) topoisomerase IIa (TOPO2A) and IIb (TOPO2B) mRNA expression in HOS-parental, HOS-R/DOXO Drug-ON and Drug-OFF by Western blot (WB bands and the Ratio DHFR/Actin represented by the Y axis and the Ratio Resistant/Parental DHFR expression values showed above corresponding column);. ND—Not done; R—Resistant; ETOP—Etoposide; CISP—Cisplatin; MAF—Mafosfamide; VCR—Vincristine. *p* < 0.05 was considered statistically significant (the values presented in the Figure 1A,B were considered statistically significant).

In order to establish resistant cell lines, a panel of six osteosarcoma cell lines was exposed to increasing concentrations of methotrexate (MTX) or doxorubicin (DOXO). Parental and resistant lines were assessed for their response to chemotherapy in terms of cell viability, migration ability, and resistance index. Acquired resistance to MTX developed in 5/6 lines (83%) up 1 µM for HOS, Saos-2, Saos-2-B and 143B after 3 months, and up to 0.03 µM after nine months exposure for MG-63 (Figure 1A). Half-maximal inhibitory concentration (IC50) of sensitive parental (MTX-IC50 range 0.04–0.05 µM) and resistant lines (MTX-IC50 range 1.9–6 µM) are given in Table S1. The IOR/OS18 cell line exhibited the highest IC50 value (1.3 µM) that could correspond to an intrinsic resistance to MTX. No further resistance to MTX was obtained. Acquired resistance to DOXO was obtained in only one cell line (HOS, 17%) after four months exposure of increasing concentrations up to 1.3 µM (Figure 1B). Under continuous exposure to increasing concentrations of mafosfamide (MAF) up to two months none of the six cell lines were able to survive.

No differences in morphology, growth rate or migration ability were detected between the MTX-resistant cell lines and their parental counterparts (Figure S1). In contrast, HOS-R/DOXO grew and migrated more slowly than its parental line (doubling time 45 h versus 25 h; after 40 h migration, doubling time was around 70% versus 20%, respectively) (Figure S1).

We then evaluated the stability of this acquired chemotherapy resistance by culturing resistant lines with or without the compound for at least nine weeks, and determined the resulting IC50 values. Under continuous drug pressure (Drug-ON) all lines exhibited high Resistance Index (RI) values for MTX (>37), while Drug-OFF cell lines exhibited variable behavior (Figure 1A). HOS-R/MTX, 143B-R/MTX and Saos-2-R/MTX maintained RI at a similar level, RI of Saos-2-B-R/MTX and HOS-R/DOXO decreased, whereas MG-63-R/MTX RI nearly normalized in two weeks (Figure 1A).

### 2.2. Cross-Resistance to Other Drugs

The possibility of a cross-resistance to other compounds was evaluated in resistant cell lines in Drug-ON and Drug-OFF culture conditions (Figure 1A,B). Cells were exposed to different concentrations of etoposide (ETOP), cisplatin (CISP), DOXO, MTX, MAF, and vincristine (VCR) or to the multi-tyrosine kinase inhibitor cabozantinib (Cabo) for 72 h before cell viability evaluation and RI calculation.

Cross-resistance was not detected with any tested compound in the MTX-resistance lines Drug-ON or Drug-OFF, except for MG-63-R/MTX (Figure 1A). Surprisingly, in Drug-OFF culture conditions, the MG-63-R/MTX exhibited a five- to 10-fold increased RI for MAF, CISP, and ETOP (Figure 1A).

For HOS-R/DOXO, cross-resistance was detected with ETOP (RI = 276), VCR (RI = 172), and MTX (RI = 12) (Figure 1B). The co-treatment with the PgP inhibitor verapamil almost abolished the resistance to DOXO and VCR (Figure 1C), but not to ETOP or MTX (data not shown), suggesting the involvement of a PgP (MDR1/ABCB1) multi-drug resistance phenomenon. The weaker PgP inhibitor, cabozantinib, at a dose that did not impact cell survival, did not modify the RI of any drug tested (data not shown). A strong increase in MDR1 mRNA levels was detected by RT-qPCR in the resistant HOS-R/DOXO cells compared to the parental line (Figure 1D). A decrease in topoisomerase-II (TOPO2A) protein level was detected by Western-blot in the R/DOXO Drug-ON and Drug-OFF cells compared to the parental line (Figure 1E).

### 2.3. Copy Number and Gene Expression Differential Analysis between Resistant and Parental Lines

Based on copy number abnormalities (CNA) by Oligonucleotide Comparative Genetic Hybridization Array (aCGH) and gene expression (GE) profiles by RNA sequencing (RNAseq), the resistant lines clustered according to their genetic background rather than the resistance mechanisms (clustering analysis) (Figure S2)

**Figure 2:**
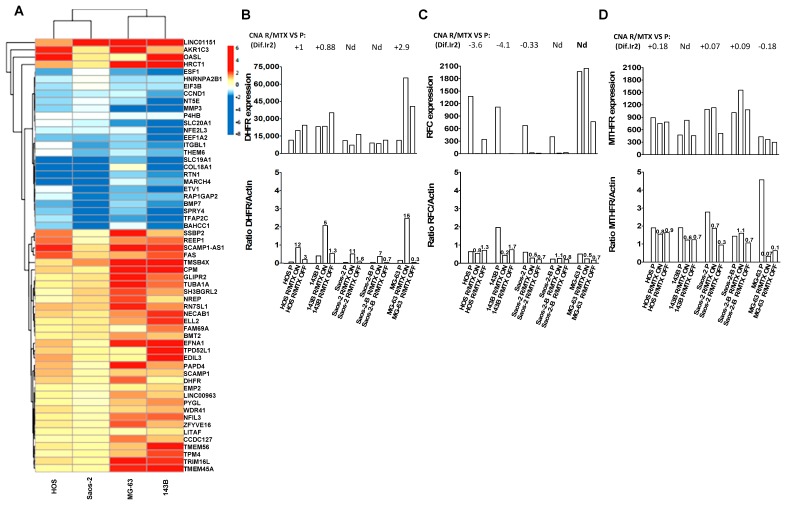
Differential analysis of the MTX-resistant and parental cell lines. (**A**) Heatmap illustrating color-coded expression levels of the most differentially expressed genes from RNA sequencing of MTX-resistant Drug-OFF versus parental cell lines; (**B**) dihydrofolate reductase (DHFR) modification in MTX-resistant compared to parental cell lines at Copy Number Abnormalities (CNA) obtained by aCGH, mRNA expression (RNA sequencing), and protein level (Western blot—Ratio DHFR/Actin represented by the Y axis and the Ratio Resistant/Parental DHFR expression values showed above corresponding column); (**C**) reduced folate carrier (RFC/SLC19A1) modification in MTX-resistant compared to parental cell lines at Copy Number Abnormalities (CNA; aCGH), mRNA expression (RNA sequencing), and protein level (Western blot); (**D**) methylenetetrahydrofolate reductase (MTHFR) modification in MTX-resistant compared to parental cell lines for Copy Number Abnormalities (CNA; aCGH), mRNA expression (RNA sequencing), and at protein level (Western blot).

Comparison of CNA-acquired changes of each MTX-resistant line to their respective parental counterpart showed common acquired CNA in MTX-resistant lines issued from similar genetic backgrounds (HOS/143B, Saos-2/Saos-2-B and MG-63, respectively), in regions which appeared to contain genes involved in MTX metabolism and resistance. We further analyzed the differential expression of these genes by RNAseq between each parental and corresponding MTX-resistant line Drug-ON and Drug-OFF and validated some findings (dihydrofolate reductase (DHFR) and reduced folate carrier (RFC/SLC19A1)) at protein level (Figure 2A–C).

Chromosome 5q region containing Dihydrofolate reductase (DHFR) (chr5:80,626,228-80,654,983) was gained in HOS-R/MTX and 143B-R/MTX, amplified in MG-63-R/MTX, and not modified in the other lines compared to their parental counterpart (Figure S3). While under MTX pressure increased mRNA gene expression (GE) and protein (Western Blot - WB) levels were seen in all five resistant lines. In Drug-OFF condition, the mRNA GE level of DHFR remained increased while the protein level decreased close to the parental level (Figure 2A,B). Compared to their parental line, HOS-R/MTX and 143B-R/MTX also lost regions in chromosome 2q containing UGT1A (UDP glucuronosyltransferase family 1 member A) (chr2: 233,585,439-233,773,299) and in chromosome 21q containing Reduced Folate Carrier gene (SLC19A1/RFC) (chr21: 46,934,628-46,962,385) and COL18A1 (chr21: 45,405,137-45,513,720). The 2q and 21q region losses were not observed in any other resistant line. No SLC19A1/RFC inactivating mutation was detected (RNAseq analysis). However, the mRNA GE level of SLC19A1/RFC was decreased in all MTX-resistant lines irrespective of the drug pressure (Drug-ON/Drug-OFF conditions), although in MG-63-R/MTX, the SLC19A1/RFC mRNA level remained high. This was confirmed at protein level, except for HOS and Saos2-B MTX-resistant lines where protein level was similar to the parental lines (Figure 2C). The MTHFR-containing region (chr1:564,423-17,221,943) was gained only in the resistant Saos-2-R/MTX and Saos-2-B-R/MTX cells, no mRNA level change was observed in any of the five MTX-resistant lines, and the protein level was slightly decreased in MTX-resistant Saos-2 but stable in Saos-2-B, and massively decreased in MG-63 MTX-resistant line (Figure 2D).

To explore other potential mechanisms of resistance common to all MTX-resistant lines, we performed a differential GE analysis between all MTX-resistant lines Drug-ON versus their parental counterpart, and Drug-OFF versus their parental counterparts (Figure 2). Differential genes implicated in resistant compared to parental lines were functionally analyzed with ToppGene. In Drug-ON conditions, the most significant enriched biological process was cell migration (GO:0016477), including MMP3 which harbored the highest significant negative fold change (log2 Fold Change −3.85; adjusted *p* value 1,60×10^-15^). In Drug-OFF conditions compared to parental lines, the most significant enriched pathway was histone acetyltransferase (HAT; ID1270435). Among the 100 most significant fold changes, we found genes implicated in osteosarcoma oncogenesis (RTN1) [7], in more general mechanisms of chemotherapy resistance such as up-regulation of several genes implicated in transcription (ESF1, CCND1, NFE2L3, ETV1, NFIL3) and in the MAPK pathway (EFNA1, TPD52L1, EDIL3). However, there were also gene changes that suggested less metastatic potential (up-regulation of Fas [8], down-regulation of MMP3) and enhanced apoptosis (up-regulation of Fas, down-regulation of EIF3B) [9,10]. Enrichment in genes located on chromosome 5q14 was also observed (SCAMP1, SCAMP1-AS1, SSBP2, TENT2, ZFYVE16, EDIL3). SSBP2 (chr5:81,413,021-81,751,797) participates in DNA damage response and maintenance of genome stability and has a role in the telomeres protection [11].

**Figure 3:**
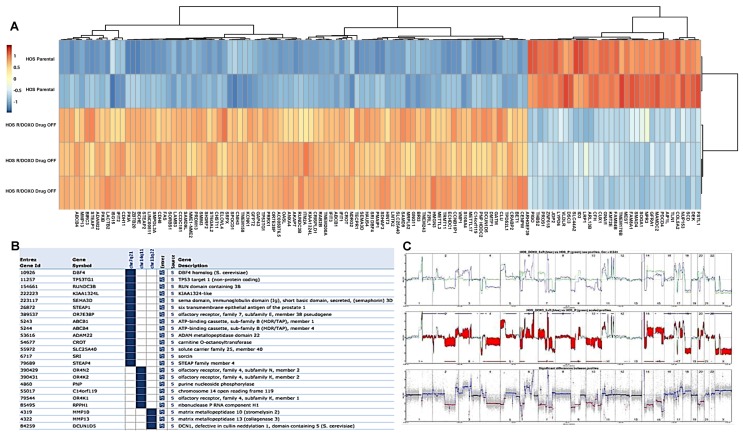
Differential analysis of the DOXO-resistant and parental cell lines. (**A**) Heatmap illustrating color-coded expression levels of the most differentially expressed genes from RNA sequencing of HOS-R/DOXO Drug-OFF (*n* = 3) versus parental cell line (*n* = 2); (**B**) genes found significantly enriched for specific chromosomal regions (cytobands); (**C**) direct comparison of HOS-R/DOXO versus HOS-parental CNA profiles. Upper panel: unscaled CNA profiles for HOS-R/DOXO (blue) and HOS-parental (green). Middle panel: same profiles after dynamics scaling of the HOS-parental profile, with significant differences colored in red areas, with corresponding segment positions as blue or red bars. Lower panel: segmentation of the difference profile.

In the unique doxorubicin-resistant line HOS-R/DOXO, the acquired gained CNA translated in an increased mRNA GE level (Figure 3). The most significantly gained regions in HOS-R/DOXO compared to parental line were on chromosomes 7:86,259,619-88,276,590 (Diff.l2r + 4.7369) containing ABCB1/MDR1 and ABCB4, and 11:102,449,766-103,152,951 (Diff.l2r + 3.1626) containing several matrix metalloproteinases (MMPs) (Figure 3A). GE analysis showed an enrichment (Figure 3B) in genes located on the same regions: on chromosome 7q21 (FZD1, CLDN12, DMTF1, STEAP1, SRRT, GNAI1, GNG11, ADAM22, PEG10) and 7q21.12 (ABCB1/MRD1, KIAA1324L, TMEM243, STEAP4, SLC25A40, RUNDC3B) and on chromosome 11q22.3 (ACAT1, MMP20, DCUN1D5, GRIK4, MMP1, MMP3, MMP8, MMP10, MMP12, MMP13, KDELC2, RAB39A, PTS, ELMOD1). Moreover, the cytoband 6p21.3 enriched in genes involved in antigen processing (e.g. human leukocyte antigen - HLA) and presentation (GO:0019882: HLA-C, HLA-DMB, HLA-DPA1, MICB, HLA-DRB5, HLA-F, PSMB8, PSMB9, TAPBP, BAG6). The highest significant positive fold changes were observed for ABCB1/MDR1 (log2 Fold Change +6.49; adjusted *p* value 6.63×10^-100^) followed by MMP13 (log2 Fold Change +6.40; adjusted *p* value 5.35×10^-88^). Several other regions with acquired CNA containing multiple multidrug resistance genes were also up-regulated in the resistant line in GE (Figure 3C). Chr7q21.3-q32.3 loss comprises the alpha/beta hydrolase MEST (Mesoderm-specific transcript) which has the highest significant negative fold change (log2 Fold Change −9.94; adjusted *p* value 8.78×10^-173^) and has been implicated in osteosarcoma oncogenesis [12,13]. Decreased mRNA expression of RFC and increased mRNA expression of DHFR, although not significant, might have participated in the slight MTX resistance observed in the DOXO-resistant line (Figure 1B). One cancer stem cell marker SOX2 was up-regulated in HOS-R/DOXO compared to its parental line (log2 FoldChange 1.89, adjusted *p* value 6,45×10^-04^), while none of the cancer stem cell markers (SOX2, OCT4, SSEA4, NANOG and ABCG2) [14] were modified in MTX-resistant lines.

These drug-induced changes suggest a broader impact of the acquired resistance in tumor cell behavior, especially in an in-vivo context.

### 2.4. In-Vivo Primary Tumor Characteristics of HOS and Saos-2-B Bioluminescent Orthotopic Parental and MTX and DOXO Resistant-CDX Models

To explore the impact of the in-vitro acquired resistance on the in-vivo tumor behavior, the parental and resistant HOS and Saos-2-B lines stably transduced with Luc/mKate2 vector to express luciferase (above 90% Luc/mKate2 positive cells) (Figure S4) were injected into NSG mice at an orthotopic (intratibial) site. Primary tumor growth, bone morphology changes and metastatic dissemination were followed using an IVIS SpectrumCT system (bioluminescence and CT scan).

Bone engraftment rates of HOS-R/MTX and parental counterpart were maximal (100%), whereas the HOS-R/DOXO-CDX had a slightly lower engraftment rate (66%) (Table 1).

**Figure 4:**
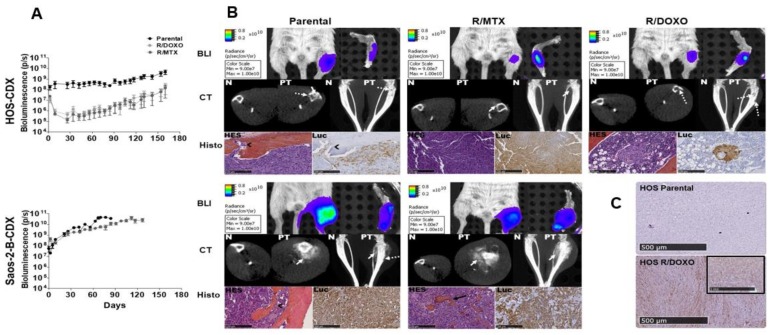
Primary tumor characteristics of the bioluminescent parental and resistant orthotopic cell-derived osteosarcoma xenograft models developed in NSG mice by intratibial injection. (**A**) Primary tumor in-vivo bioluminescence (BLI) detection overtime for parental and resistant-CDX; (**B**) orthotopic osteosarcoma bioluminescent models in NSG mice at time of sacrifice: HOS-CDX (top panel), Saos-2-B-CDX (bottom panel) (both parental-CDX, both R/MTX-CDX and HOS-R/DOXO-CDX). In-vivo BLI imaging by IVIS SpectrumCT system of the primary tumor (left leg) compared to the control leg (right leg). In-vivo CT scan imaging (CT) by an IVIS SpectrumCT system of the normal leg (N) and injected leg (PT). Primary tumor (PT), showing osteocondensation (plain white arrow) and osteolysis (dotted white arrow), changes were first noted 63, 91, and 77 days after injection for HOS-parental-CDX, HOS-R/MTX-CDX, HOS-R/DOXO-CDX and at day 41 and 49 days after injection for Saos-2-B-parental-CDX and Saos-2-R/MTX-CDX, respectively. Histology (Histo) using Hematoxylin Eosin Safranin (HES) and luciferase staining of the primary tumor and normal bone (not injected) at 7,45× magnification, showing osteoid matrix (big black arrow) and infiltration by tumor cells in the bone (small black arrow); (**C**) Immunohistochemical (IHC) staining for MDR1 protein expression in primary tumor tissue of HOS-parental and HOS-R/DOXO-CDX models. Normal human kidney was used as positive control (image surrounded with black lines).

The HOS-resistant lines had more difficulties to adapt to the in-vivo bone environment, with an initial decrease in in-vivo bioluminescence (up to 27 days), followed by a slightly faster growth compared to the HOS-parental-CDX (Figure 4A).

Bone engraftment rate of Saos-2-B-R/MTX and parental-CDX were comparable (83% versus 100%, respectively) (Table 1). Both parental and resistant models exhibited similar primary tumor growth (Figure 4A).

The different models revealed tumor-bearing tibia bone structure abnormalities similar to those observed in osteosarcoma patients.

The resistant models retained the primary tumor-induced bone abnormalities of their parental counterpart in the Computed Tomography (CT scan) (Figure 4B). The slow growing osteolytic HOS-CDX were confined to bone, while the fast growing Saos-2-B-CDX induced aggressive osseous and extraosseous masses with osteocondensation deforming the leg. HES staining confirmed the osteoblastic phenotype of all models with some fibroblastic component (Figure 4B) and no morphological differences between parental and resistant-CDX. In-vivo and ex-vivo bioluminescence, CT scan and histology (HES and luciferase staining), confirmed that the changes observed were caused by human osteosarcoma cells (Figure 4B; Table S2). PgP protein expression, was detected by IHC in HOS-R/DOXO-CDX but not in HOS-parental-CDX (Figure 4C) or HOS-R/MTX-CDX (data not shown) primary tumors. Differential analysis of the CGH profiles of the CDX models and the cell lines (In-vitro) they were issued of, showed modifications induced by the in-vivo setting in both parental and resistant models (Figure S5). Several low amplitude CNA changes were observed in the Saos-2-B-R/MTX-CDX which does not change the general CGH profile between CDX and the cell line of origin. In the HOS-R/MTX-CDX, the amplitude of the chr5 gain and chr18 loss were less important than in the cell line, but remained. The most important changes were observed between the HOS-R/DOXO-CDX and the cell line it derived of, with no CNA in region loss (chr 2q, 4p, 6p, 10) or gain (chr 3p) in-vitro; and losses of region without previous CNA (chr 6q, 3q, 13) and gain of regions without previous CNA (chr22q).

### 2.5. In-Vivo Metastatic Behavior of the Resistant Orthotopic Bioluminescent-CDX Models

**Figure 5:**
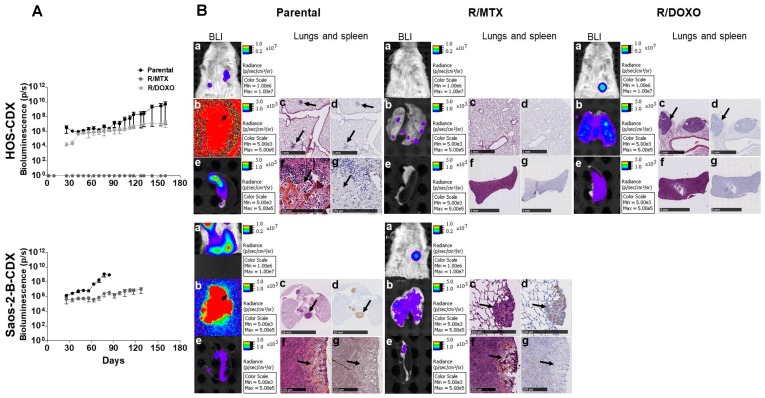
In-vivo metastatic behavior of the bioluminescent parental and resistant orthotopic cell-derived-xenografts osteosarcoma models in NSG mice by intratibial injection. HOS-CDX (top panel), Saos-2-B-CDX (bottom panel). (**A**) In-vivo bioluminescence (BLI) detection of parental and resistant-CDX metastases overtime; (**B**) CDX-models at sacrifice time: HOS- and Saos-2-B-parental-CDX, HOS- and Saos-2-B-R/MTX-CDX and HOS-R/DOXO-CDX. in-vivo *(***a**) and ex-vivo BLI of lung (**b**) and spleen (**e**) metastases. Lung Hematoxylin Eosin Safranin (HES) (**c**) and luciferase staining (**d**) at 1,5× magnification for all HOS-CDX and 0,21 and 10,8× magnification for Saos-2-B-parental-CDX and Saos-2-B-R/MTX-CDX, respectively. Spleen HES (**f**) and luciferase staining (**g**) at 10,8 and 0,36× magnification for HOS-parental-CDX and both resistant-CDX, respectively, and 3x for all the Saos-2-B-CDX models. Arrows show metastases.

Metastatic foci were detected by in-vivo bioluminescence in all CDX models as early as 30 days after intratibial injection (Figure 5A) except for HOS-R/MTX-CDX where metastases were detectable only by ex-vivo bioluminescence (Figure 5B-top-panel). Metastases in parental-CDX models grew faster than in resistant-CDX models, without correlation with the primary tumor growth rate and size (Figure 5A). At sacrifice (days 84 and 127 for parental and Saos-2-B-R/MTX-CDX, respectively, and day 160 for all HOS models), combined ex-vivo bioluminescence and histology confirmed lung metastases in all models (Figure 5B; Table 1). Lung metastases were bigger and more frequent in Saos-2-B-parental-CDX than in HOS-parental-CDX, and in parental-CDX compared to their resistant-CDX counterparts. Unique bone metastases on the opposite leg (not injected) and unusual spleen metastases were detected in all models except in HOS-R/MTX-CDX. HES did not detect morphological differences between parental and resistant-CDX (Figure 5B). Metastases were not detected by CT scan due to its resolution, however HES staining confirmed the osteosarcoma nature of metastases detected by bioluminescence (Figure 5B; Tables S2 and S3.

### 2.6. In-Vitro Secondary Cultures Issued from CDX Models

After mice sacrifice, cells isolated from primary tumors of each CDX model (no in-vivo treatment was performed) were cultured for two in-vitro passages. All resistant-CDX-derived cells grew in-vitro. Drug response was assessed. RI of in-vitro resistant-CDX-derived cells were either lower (HOS-R/DOXO-CDX-cells RI = 224 and 42, respectively), higher (HOS-R/MTX-CDX-cells; RI = 150 and >2500), or stable (Saos-2-B-R/MTX-CDX-cells; RI = 38 and 34) compared to the matched RI of the in-vitro initial resistant cells (Table 2).

## 3. Discussion

Resistance to chemotherapy and metastatic phenotypes are the two main problems in osteosarcoma patients that lead to recurrence and death. We developed six new in-vitro osteosarcoma models, resistant either to MTX (HOS, 143B, MG-63, Saos-2, and Saos-2-B cell lines) or DOXO (HOS), by continuous in-vitro drug exposure, adding new models to those previously described [2,15,16,17,18,19,20]. We established and characterized orthotopic-CDX models in-vivo, derived from these resistant lines to compare their in-vivo behavior (primary tumor growth/metastatic potential) to their parental counterparts.

High level of in-vitro acquired resistance was obtained with MTX (5/6 cell lines), while only one of the six lines developed resistance to DOXO. This is consistent with observation in other models derived from patient samples which showed resistance to MTX while still sensitive to DOXO and CISP [14]. Continuous in-vitro exposure to the drug induced large CNA changes translated at transcriptomic GE mRNA level, in a drug and cell line dependent manner [21]. These drug-induced chromosomic/transcriptomic bloc changes included regions with genes known to be involved in DOXO or MTX resistance. Additional molecular and cellular programs not directly linked to the mechanism of action, and metabolism of one drug was modified on acquired resistant lines. They corresponded to deregulated transcriptional programs involved in more general tumor behavior such as invasion and metastatic potential.

For the unique, highly DOXO-resistant line HOS-R/DOXO line (RI = 224), we observed a multi-drug resistant phenotype, with cross-resistance to other agents used in osteosarcoma treatment and substrates of PgP (e.g., ETOP, VCR, but also to MTX), associated with ABCB1/MDR1 gain translated in high MDR1 mRNA and PgP protein expression. The DOXO-resistant line gained several chromosomic regions associated with mRNA expression up-regulation on the chromosome 7 region containing ABCB1/MDR1 and ABCB4, and several other regions encoding various multiple multi-drug resistance genes or genes implicated in the apoptotic response to doxorubicin. Up-regulation of MDR1 has been associated with chemo-resistance development of osteosarcoma tumor-initiating cells [22], and SOX2 mRNA, a marker of cancer stem cells, was up-regulated in our DOXO-resistant line. The multidrug-resistant phenotype was partially reverted in-vitro by the PgP inhibitor verapamil, as shown in other osteosarcoma resistant lines with other PgP inhibitors [23,24,25]. A third of osteosarcoma patients expressed PgP at diagnosis [26]. ABCB1/MRD1 inhibitors are being explored in clinical trials [27,28]. Another potential weaker PgP inhibitor and studied in osteosarcoma relapse treatment (NCT02243605) [29], the multi-tyrosine kinase inhibitor cabozantinib, did not revert PgP phenotype when used at a dose that did not inhibit cell proliferation, conversely to what was shown in hepatoblastoma cells [30].

For our MTX-resistant lines, high level of resistance was obtained (RI > 37) and persisted after drug removal although at lower level, except for MG-63 which lost MTX-resistance. Higher resistance has been observed in patient-derived cell lines [31]. All our MTX-resistant cell lines Drug-ON exhibited changes in the ubiquitous transporter for folates SCL19A1/RFC and the MTX target DHFR, although by different mechanisms. SCL19A1/RFC mRNA GE was down-regulated in all MTX-resistant cell lines, associated with decreased protein level in all lines except HOS-R/MTX and Saos-2-B-R/MTX, irrespective of CNA loss (observed in HOS and 143B but not in the other MTX-resistant lines). DHFR mRNA and protein expression was up-regulated in all MTX-resistant cell lines under drug pressure. Up-regulation of DHFR mRNA and protein expression was previously associated with the development of chemo-resistance of osteosarcoma tumor-initiating cells [22]. MTX was also described as decreasing Saos-2 cell proliferation by S-phase cell cycle inhibition and increasing apoptosis, probably by a DHFR-mediated mechanism [31,32] as epigenetic modifier (increase of histone H3 acetylation) capable to modify some cell differentiation-related genes (e.g., COLLI, ALPL) in patient-derived cell lines. No cancer stem cell marker or cell differentiation-related genes were modulated at the mRNA level in our MTX-resistant lines. On Drug-OFF conditions, irrespective of the gain of the DHFR region (present in HOS, 143B and MG-63, but not in Saos-2 or Saos-2-B MTX-resistant lines) and despite persistent up-regulated mRNA levels and increased resistance index, protein levels were decreased, suggesting DHFR post-transcriptional regulation and the involvement of possible other resistance mechanisms. The link between RB1 expression and MTX-resistance mechanisms previously reported with increased DHFR expression by gene amplification in RB1-expressing osteosarcoma cell lines and RFC expression decrease without DHFR involvement in RB1 deficient (not expressing) lines [15], did not fully apply to our models. MTX-resistance persisted despite drug removal, although at lower level for HOS-R/MTX, but was lost in MG-63-R/MTX within two weeks without drug. In MTX-resistant lines no in-vitro cross-resistance was detected with the other drugs tested, as opposed to cross-resistances reported (doxorubicin, ifosfamide, epirubicine, theprubicin, or paclitaxel) in low/intermediate MTX-resistant Saos-2 lines (RI of 5 and 13, respectively) with low SCL19A1/RFC expression [33]. The only exception was for MG-63 Drug-OFF that lost MTX-resistance but acquired low levels of resistance to other drugs, suggesting other resistance mechanisms than those involved in MTX-transport and mechanism of action.

Several more general cellular and biological pathways were modulated in the chemo-resistant lines related to cell adhesion/motility, extracellular matrix organization/degradation/composition, and cellular microenvironment. These processes, not fully assessed in-vitro, might affect the resistant cell behavior in-vivo. In DOXO- and MTX-resistant osteosarcoma cells, Fas expression was increased, suggesting a decreased in-vivo metastatic potential [8]. Similarly, MMP3 decrease in MTX-resistant cells suggested less invasive potential [34,35]. In our in-vitro resistant models, no modification of cancer stem cell markers was observed as it has been seen in PDX models [14]. We also observed in the DOXO-resistant line compared to its parental counterpart an enrichment of the HLA cluster genes on cytoband 6p21.3. The amplification of 6p21 cytoband is present in around 15% of OS at diagnosis and link to a poorer survival [36] and might appeared in matched diagnosis primary tumors/metastatic relapse samples [37]. Doxorubicin is known to be an immunogenic drug [38]. In addition, several other chromosomic modifications observed in our resistant models have been observed at diagnosis in patient samples and associated with poor outcome (chr13q LOH withRB1 suppressor gene [36,39,40]; chr3q loss with gene LSAMP tumor suppressive [39,40], chr6q loss [40]). In addition, the bone microenvironment is known to have a key role in osteosarcoma progression [41], and has been shown to influence drug sensitivity in osteosarcoma syngeneic models [42] and might influence resistance phenotype [21].

We developed in-vivo orthotopic intratibial bioluminescent parental and resistant-CDX models in NSG mice, with the experimental procedure used previously [43]. The different general primary bone tumor behavior (slow growing osteolytic HOS-CDX, fast growing osteocondensed Saos-2-B-CDX), metastatic potential (faster metastatic spread in Saos-2-B-CDX than in HOS) and morphology [43] were retained by the respective resistant models. However, all resistant-CDX had a slower and lower lung metastatic spread than parental-CDX. Similar behavior has been observed with other in-vivo models of metastatic spread by direct intravenous injections of doxorubicin-resistant osteosarcoma U2OS and Saos-2 variants (MDR1 overexpression by gene amplification) in athymic nude mice, when resistant cells were injected straight after in-vitro treatment, but not when cultured in a drug-free medium for a week before injection [44]. This contrasts with the observation in patients with osteosarcoma, where high Pgp expression is observed in around 10% of them and seems correlated with metastasis development and poor response to pre-operative chemotherapy [26]. In our CDX models, secondary cultures of the resistant lines after sacrifice retained their resistant phenotype. Time intervals between cell injection and detection of primary tumor growth and metastatic spread are still compatible with drug testing in-vivo.

Our resistant-CDX models can substantially help to evaluate new drug efficacy in osteosarcoma and complement the few pre-existing osteosarcoma models. Future work will include the comparison of our models to the PDX models that we are currently generating issued from relapsed osteosarcoma and the human relapsed osteosarcoma cohorts that might be available in the future (several molecular profiling programs ongoing).

## 4. Materials and Methods

### 4.1. Cells Culture

Human osteosarcoma cell lines HOS, 143B, Saos-2, Saos-2-B, MG-63 and IOR/OS18 (Table S4) with different genetic background [45,46,47] were cultured in Dulbecco’s modified Eagle medium (DMEM, Invitrogen, Saint Aubin, France) supplemented with 10% (*v*/*v*) fetal bovine serum (FBS, Invitrogen, Saint Aubin, France) at 37 °C in a humidified atmosphere (5% CO_2_ and 95% air), under mycoplasma free conditions.

### 4.2. Compounds

Doxorubicin (DOXO), methotrexate (MTX), cisplatin (CISP), etoposide (ETOP), vincristine (VCR), and verapamil (VER) were purchased from Sigma Aldrich (Lyon, France), mafosfamide (MAF) from Toronto Research Chemicals Inc. (Toronto, OA, Canada), and cabozantinib (Cabo) from LC Laboratories (Woburn, Canada). All compounds were solubilized in dimethyl sulfoxide (DMSO; Sigma Aldrich, Lyon, France), except cisplatin, solubilized in N, N-dimethylformamide (DMF; Sigma Aldrich, Lyon, France), at 10 mM stock solutions, and stored at −20 °C.

### 4.3. In-Vitro Development of Chemo-Resistant Osteosarcoma Cell Lines

Cells were seeded into 6-well plate at 100,000 cells/well (HOS, 143B, MG-63) or 120,000 cells/well (IOR/OS18, Saos-2, Saos-2-B) in DMEM supplemented with 10% FBS, then exposed continuously to an initial concentration of 0.01 µM DOXO or 0.07 µM MTX. In parallel, vehicle (DMSO) treated cells (parental) were seeded at the same conditions and used as controls. The medium was changed twice a week, and passages (1:10) were performed when cells had reached 80% confluence. The MTX concentration was progressively increased up to 0.3 µM for MG-63 and 1 µM for all other lines; DOXO concentration was raised up to 1.3 µM for HOS. The resistant lines were maintained in culture with the maximal tolerated drug concentration and named as “Drug-ON” cell lines. Drug pressure was stopped after resistance confirmation, and resistant cells were cultured in drug free medium for minimum nine weeks, designed as “Drug-OFF” cell lines. The same experiments were performed with MAF without success (treatment: initial concentration of 2.5 µM for minimum 2 months).

The cell lines that did not developed resistance, died under drug continuous exposure. Therefore, no experiments could be done.

### 4.4. In-Vitro Cell Proliferation and Cell Viability Assays

Parental and resistant derived HOS, 143B, MG-63 and IOR/OS18 lines were seeded at 5000 cells/well, and cells derived from Saos-2 and Saos-2-B at 10,000 cells/well in a 96-well plate in DMEM supplemented with 10% FBS for both assays.

Cell proliferation rate and doubling time were assessed using the IncuCyte live-cell imaging system (Essens Bioscience, Birmingham, UK). Phase-contrast photographs were collected for 72 h.

The day after seeding, cells were incubated in the presence of a range of drug concentrations for 72 h (from 0 to 100 μmol/L for DOXO, ETOP, MAF and Cabo; 0 to 50 μmol/L for CISP; 0 to 500 μmol/L for MTX; and 0 to 10 μmol/L for VCR). Verapamil was used at 5 μmol/L and cabozantinib at 0.1 μmol/L, to revert PgP function. Cell viability was evaluated using the CellTiter 96 Aqueous One Solution Cell Proliferation Assay (MTS assay) (Promega, Charbonnieres, France), according to the manufacturer instructions. The half-maximal inhibitory concentration (IC50) was determined using the GraphPad Prism5 software (Graphpad Software Inc., California, USA).

### 4.5. Wound-Healing Assay

Cells were seeded at 10,000 cells/well (HOS, 143B, MG-63, IOR/OS18), or at 20,000 cells/well (Saos-2, Saos-2-B) in 96-well ImageLock tissue culture plates (Essen BioScience, Birmingham, UK) in DMEM supplemented with 10% FBS. The day after, cell layers were scratched with the WoundMaker™, washed once and then incubated in the presence of the indicated drugs (0.01 µM MTX, DOXO, CISP and ETOP, or 0.2 µM MAF, IC50 and 10 times IC50) or DMSO (control). The wound-healing was monitored using IncuCyte™ system for 48 h. Data analyses were performed using Graphpad Prism5 Software.

### 4.6. Transfection and Cell Transduction With Luc/Mkate2 (Transgene) In-Vitro

Parental and resistant HOS and Saos-2-B cells were stably transduced with Luc/mKate2 vector as previously described [43].

### 4.7. Orthotopic Bioluminescent CDX Models

Animal experiments were approved by the CEEA26, Ethics Committee and the French Ministry of Research (APAFIS#1648-2015090713516480) and performed under the conditions established by the European Community (Directive 2010/63/UE).

The parental cell lines (HOS-Luc/mKate2, Saos-2-B-Luc/mKate2) and their resistant counterparts to either MTX (HOS-Luc/mKate2/MTX, Saos-2-B-Luc/mKate2/MTX) or DOXO (HOS-Luc/mKate2/DOXO) were established into 7-week-old immunodeficient NSG mice by unilateral intratibial injection (1.5 × 10^6^ cells in matrigel solution at 4 mg/mL), as previously described [43,48]. Cells were injected in a total volume of 10 μL Matrigel (Corning, Wiesbaden, Germany) solution at 4 mg/mL to avoid immediate spread in the blood circulation. Paratibial injection was performed applying a 30-G needle perpendicular to the tibia after a 0.5-cm skin incision. Before cell injection, periosteum was gently activated with the needle (periosteum denudation) to ensure bone development of the primary tumor. Buprenorphine at 0.3 mg/kg was applied in addition to the general anesthesia (3% isoflurane).

Mice were monitored clinically every week. At sacrifice, xenografts were collected and processed for histological analyses or snap-frozen in liquid nitrogen and stored at −80°C for subsequent RNA extraction. Some resistant-CDX tumors were collected, mechanically dissociated, and cultured in-vitro (secondary culture) in DMEM supplemented with 10% FBS.

### 4.8. In-Vivo and Ex-Vivo Computed Tomography (CT) Scan and Bioluminescence (BLI) Imaging

In-vivo and ex-vivo images were acquired using IVIS SpectrumCT (Perkin Elmer, Courtaboeuf, France) as previously described [43]. Briefly, NSG mice under anesthesia (3% isoflurane) were injected intraperitoneally with 150mg/kg D-luciferin (Beetle luciferin, Promega, Charbonnieres, France). Whole mice bodies were imaged for primary tumor and metastases detection (BLI and CT scan). After sacrifice, legs, lungs, and spleen were immersed in 150 µg/mL D-luciferin and imaged individually. Organs were then washed in PBS and fixed in a 4% paraformaldehyde before paraffin embedding.

### 4.9. Histological Analysis and Immunohistochemistry (IHC)

Formaldehyde-fixed-paraffin-embedded (FFPE) tissue sections (4 µm) were either stained with hematoxylin-eosin-safranin (HES) for morphology, or processed for immunohistochemistry as described before [43]. Primary antibodies: mouse anti-firefly luciferase monoclonal antibody (1:200, ThermoFisher Scientific, Massachusetts, USA) and mouse monoclonal anti-human MDR1 antibody (1:20, Merck Millipore, Fontenay-sous-Bois, France). Slides were scanned using NanoZoomer 2.0-HT (Hamamatsu Photonics, Massy, France). Histology was reviewed by a bone pathologist. Normal human kidney and IGR-N91-Luc-neuroblastoma cells [49] were used as positive controls for MDR1 and luciferase staining, respectively.

### 4.10. Nucleic Acid Extraction

DNA and RNA from cells and CDX samples were isolated using AllPrep DNA/RNA mini kit (Qiagen) according to manufacturer’s instructions. Quantification/qualification were performed using Nanodrop 2000 spectrophotometer (Thermo Fisher Scientific, Massachusetts, USA) and Bioanalyzer DNA 7500 (Agilent, California, USA).

### 4.11. Oligonucleotide Comparative Genetic Hybridization Array (aCGH) Assay

Sex-matched normal DNA from a pooled human DNA (Promega, Charbonnieres, France) was used as a reference. Oligonucleotide aCGH processing was performed according to manufacturer’s protocol version 7.5 (http://www.agilent.com). 500 ng of tumor and reference DNAs were fragmented with AluI and RsaI (Euromedex, Souffelweyersheim, France) and labeled with Cy5-dUTP and Cy3-dUTP, respectively. Hybridization was carried out on SurePrint G3 Human CGH Microarray 4 × 180K (Agilent Technologies, California, USA) arrays for 24 h at 65 °C in a rotating oven (Robbins Scientific, CA, USA) at 20rpm and followed by appropriate washing steps. Microarrays were scanned with an Agilent G2505C DNA Microarray scanner at 100% PMT with 3 µm resolution at 20 °C in low ozone concentration environment. Data were extracted using the Feature Extraction software (v11.5.1.1, Agilent), along with protocol CGH_1105_Oct12. All further data manipulations were performed under the R statistical environment (v3.4, http://cran.r-project.org). Raw intensities were normalized according to their dye composition (Cy3 fitted over Cy5). Data were transformed to log2(Test/Ref) and normalized according to their local GC content through a lows regression. Resulting profiles were segmented with the CBS algorithm [50] implemented in the DNAcopy package (v1.42) using default parameters. Profiles were centered to the most centered out of the three most populated peaks of the log2(Test/Ref) distribution density. Aberrations were called using a threshold defined as one-fourth of the median value of the absolute differences between consecutive log2(Test/Ref) measures along the genome. Profiles were then aggregated and hierarchically clustered (Pearson distance, Ward aggregation method). Pair-wise comparisons of profiles were performed, first applying a linear regression of the profile with the lowest dynamics (measured as its interquartile range) to the profile with the highest one; the probe-based difference of the log2 (Test/Ref) of the two profiles was then computed, then was segmented and called as described previously. Genomic regions called as different in the differential profiles were annotated using UCSC annotation tables (cytoBandIdeo, cpgIslandExt, wgRna, refGene, dgvMerged) for the hg19 genome build.

### 4.12. RNA Sequencing (Rnaseq)

RNAseq analysis was performed as previously described [51]. RNAseq libraries were prepared with a TruSeq Stranded mRNA kit following recommendations: the key steps consisted of PolyA mRNA capture with oligo dT beads 1µg total RNA, fragmentation to approximately 400 pb, DNA double strand synthesis, and ligation of Illumina adaptors amplification of the library by PCR for sequencing. Libraries sequencing were performed using Illumina sequencers (NextSeq 500 or Hiseq 2000/2500/4000) in 75 bp paired-end mode. Quality of stranded pair-ended RNAseq libraries was evaluated with fastqc (https://www.bioinformatics.babraham.ac.uk/projects/fastqc/). Reads were mapped with Salmon v0.8.1 [52] using GRCh37 ENSEMBl mRNA dataset as reference sequences. Differential mRNA expression was measured with DESeq2 R package from raw read count table [53].

Differential mRNA expression lists were compared using Venny diagram produced by Venny 2.1.0 (http://bioinfogp.cnb.csic.es/tools/venny/). Toppfun website was used for functional enrichment analysis (https://toppgene.cchmc.org/enrichment.jsp).

### 4.13. Reverse Transcription-Quantitative PCR (RT-qPCR)

Total RNA (1 µg) was reversely transcribed into cDNA using M-MLV Reverse Transcriptase (ThermoFisher Scientific, Massachusetts, USA), according to the manufacturer’s recommendations. Amplifications monitored with StepOnePlus PCR System (Applied Biosystems, Villebon-sur-Yvette, France), were performed using Maxima SYBR Green/ROX qPCR kit (ThermoFisher Scientific, Massachusetts, USA), and with a first step at 95 °C for 10 min followed by 40 cycles with 95 °C for 15 s and 60 °C for 1min. Melting curve was performed at the end of the PCR (95 °C for 15 s, 60 °C for 1 min and 95 °C for 15 s) to identify unique PCR products. The sequences of the specific primers are described in Table S5. Glyceraldehyde-3-phosphate dehydrogenase (GAPDH) from Invitrogen was used as standard. Data were analyzed by the relative quantification method using the 2–ΔΔCt formula [54].

### 4.14. Western-Blot (WB)

Parental and resistant Drug-ON and Drug-OFF cell lines were seeded into 100*20mm culture dish at 130,000 cell/dish in DMEM supplemented with 10% FBS. Cells were collected at 80–90% confluence, followed by a PBS wash and re-suspended in lysis buffer (TNEN 5mM buffer add protease inhibitor pill, NaF, and Orthovanadate) and stored at −20 °C. Proteins were extracted and measured with Pierce BCA Protein Assay Kit (ThermoFisher Scientific, Massachusetts, USA).

Western-blot analysis was performed using specific primary antibody against topoisomerase-II alpha/beta (TOPO2) (1:1000, anti-topoisomerase-II alpha + topoisomerase-II beta Antibody), anti-dihydrofolate reductase (DHFR) (1:1000), anti-reduced folate carrier (RFC) (1/200), anti-methylenetetrahydrofolate reductase (MTHFR) (1/1000), all purchased from abcam (Paris, France) and β-Actin (13E5) Rabbit mAb (1:1000, Cell Signaling Technology, Schuttersveld, Netherlands). Appropriate secondary antibodies (Cell signaling) at 1:5000 dilution were used, followed by visualization with the enhanced chemiluminescence ECL reagent (ThermoFisher Scientific, Massachusetts, USA) and imaged with ChemiDoc™MP image system (Bio-Rad, California, USA). Signal intensities were quantified with the Image Lab version5 (Bio-Rad, California, USA).

### 4.15. Statistical Analysis

Data were shown as the mean ± standard error of mean (SEM) of three independent experiments performed using Graphpad Prism^®^5 Software (Graphpad Software Inc., California, USA). The one-way ANOVA analysis was used to compare the groups. *p* < 0.05 was considered to indicate a statistically significant difference.

## 5. Conclusions

In-vitro acquired resistance to MTX and DOXO, induced CNA and possible epigenetic changes that involved specific but also more general mechanisms of resistance that might influence OS cell behavior in their microenvironment. These resistant-CDX models can help to evaluate new drug efficacy in osteosarcoma and complement the few pre-existing osteosarcoma models [2,15,16,17,18,19,20] despite the limitations of the established cell lines used. We are currently developing Patient-derived xenograft models from relapsed osteosarcoma samples which will bring complementary knowledge on human osteosarcoma drug resistance, while syngeneic (mice or dog) or humanized osteosarcoma models [20] might partially give access to the immunity role in osteosarcoma resistance to treatment. Multiplying different complementary models might help to better tailor drug testing in osteosarcoma.

## Figures and Tables

**Table 1:** In-vivo primary tumor engraftment and metastatic rate of the resistant (HOS-R/MTX-CDX, Saos-2-B-R/MTX-CDX and HOS-R/DOXO-CDX) and parental (HOS-parental-CDX, Saos-2-B-parental-CDX) orthotopic bioluminescent osteosarcoma cell line derived xenografts.

Luc/mKate2 Cell Line	Parental		R/MTX		R/DOXO	
	Primary Tumor	Metastases	Primary Tumor	Metastases	Primary Tumor	Metastases
HOS	5/5	5/5	8/8	4/8	4/6	4/6
Saos-2-B	5/6	5/6	4/4	4/4	-	-

**Table 2:** Resistance phenotype of the orthotopic secondary cultured resistant osteosarcoma CDX models. Drug sensitivity (IC50 and RI) in early secondary cell cultures derived from resistant-CDX models (HOS-R/MTX-CDX-cells, HOS-R/DOXO-CDX-cells, and Saos-2-B-R/MTX-CDX-cells) compared to the initial In-Vitro values. Parental and Resistant Drug-ON before introduction in mice and resistant Drug-ON cell line after injection in NSG mice and cultured In-Vitro after mice sacrifice.

Cell Lines	IC50 (µM)			RI		IC50 (µM)			RI	
	Parental	R/MTX		R/MTX		Parental	R/DOXO		R/DOXO	
	Before *	Before *	After *	Before *	After *	Before *	Before *	After *	Before *	After *
**HOS**	0.04	6.24	>100	156	>2000	0.06	11.3	2.09	212	41.8
**Saos-2-B**	0.05	1.93	2.05	37	34.2	NA	NA	NA	NA	NA

* Before or after injection of the cell lines in NSG mice. NA—Not available.

## Supplementary Materials

The following are available online at https://www.mdpi.com/2072-6694/11/7/997/s1, Figure S1: In-vitro characteristics of HOS and Saos-2-B parental and resistant cell lines to MTX and DOXO, Figure S2: Clustering analysis of the resistant and parental cell lines, Figure S3: Direct comparison of HOS-R/MTX, 143B-R/MTX, Saos-2-R/MTX, Saos-2-B-R/MTX and MG-63-R/MTX versus their respective parental CNA profiles, Figure S4: Characterization of luciferase-transfected osteosarcoma cells, Figure S5: Direct comparison of CDX models versus their respective origin cell line (in-vitro) (cell line from which they were issued of) CNA profiles, Table S1: Acquired in-vitro resistance to methotrexate (MTX) and doxorubicin (DOXO), Table S2: Morphological and histological characteristics of all osteosarcoma bioluminescent orthotopic CDX: HOS-parental-CDX, HOS-R/MTX-CDX, HOS-R/DOXO-CDX, Saos-2-B-parental-CDX and Saos-2-B-R/MTX-CDX, Table S3: Characteristics of metastases of all osteosarcoma bioluminescent orthotopic parental and resistant CDX models, Table S4: Characteristics of Osteosarcoma cell lines, Table S5: Primers used to amplify topoisomerase IIa (TOPO2A), multidrug resistance protein 1 (MDR1/ABCB1) or P-glycoprotein 1 (PgP) and multidrug resistance associated protein 1 (MRP1/ABCC1) cDNAs by quantitative real-time PCR. Glyceraldehyde 3-phosphate dehydrogenase (GAPDH) was used as control.
