# Supplementary material for: In-Vitro and In-Vivo Establishment and Characterization of Bioluminescent Orthotopic Chemotherapy-Resistant Human Osteosarcoma Models in NSG Mice
Source: Cancers (Basel). 2019 Jul 17;11(7):997. doi: 10.3390/cancers11070997 (PMC6678535; doi:10.3390/cancers11070997)
Supplement: Supplementary file 1 [file cancers-11-00997-s001.pdf]

Supplementary

Table S1: Acquired *in-vitro* resistance to methotrexate (MTX) and doxorubicin (DOXO). IC50 values of the Parental and resistant (Drug-ON and Drug-OFF) cell lines to MTX, DOXO and mafosfamide (MAF) as well as to DOXO Resistant (R/DOXO) cell line treated with etoposide. IC_{50} values were calculated using Prisma version5 using cell proliferation data after 72h of treatment. NA- not applicable, NO- not obtained

**Cell line**	**IC50 (μM)**											
	**Resistance to MTX**			**Resistance to DOXO**			**R/DOXO ETOP**			**Resistance to MAF**		
	**Parental**	**Drug ON**	**Drug OFF**	**Parental**	**Drug ON**	**Drug OFF**	**Parental**	**Drug ON**	**Drug OFF**	**Parental**	**Drug ON**	**Drug OFF**
HOS	0.04	6.00	5.73	0.05	11.20	4.39	0.65	180	90.5	12.70	No	No
143B	0.04	4.13	4.64	0.04	No	No	NA	NA	NA	14.30	No	No
Saos-2	0.05	2.05	1.78	0.05	No	No	NA	NA	NA	17.60	No	No
Saos-2-B	0.05	1.90	1.20	0.05	No	No	NA	NA	NA	20.30	No	No
MG-63	0.04	2.84	0.13	0.10	No	No	NA	NA	NA	13.30	No	No
IOR/OS18	1.30	No	No	0.18	No	No	NA	NA	NA	27.13	No	No

A

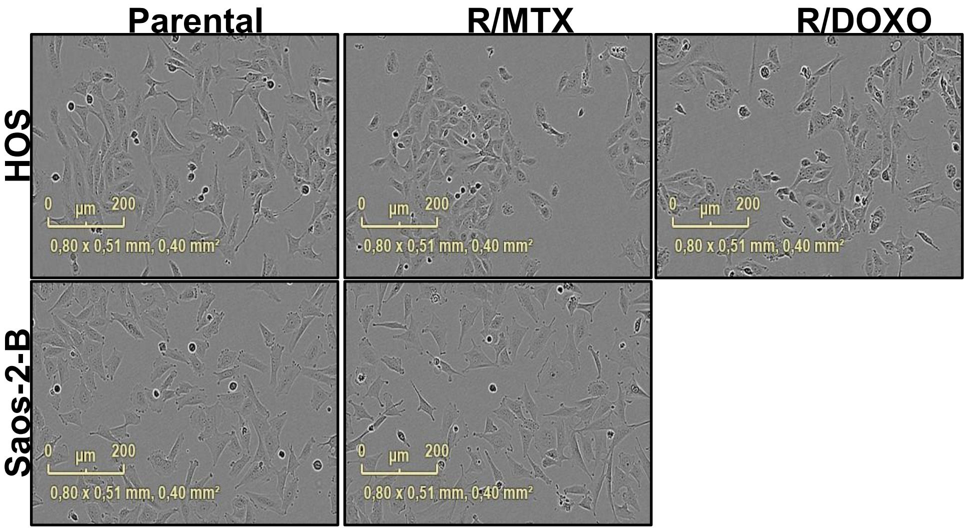

B

Cell lines	Doubling time		
	Parental (h)	R/MTX (h)	R/DOXO (h)
HOS	27	18	44
143B	15	22	-
Saos-2	32	26	-
Saos-2-B	24	29	-

C

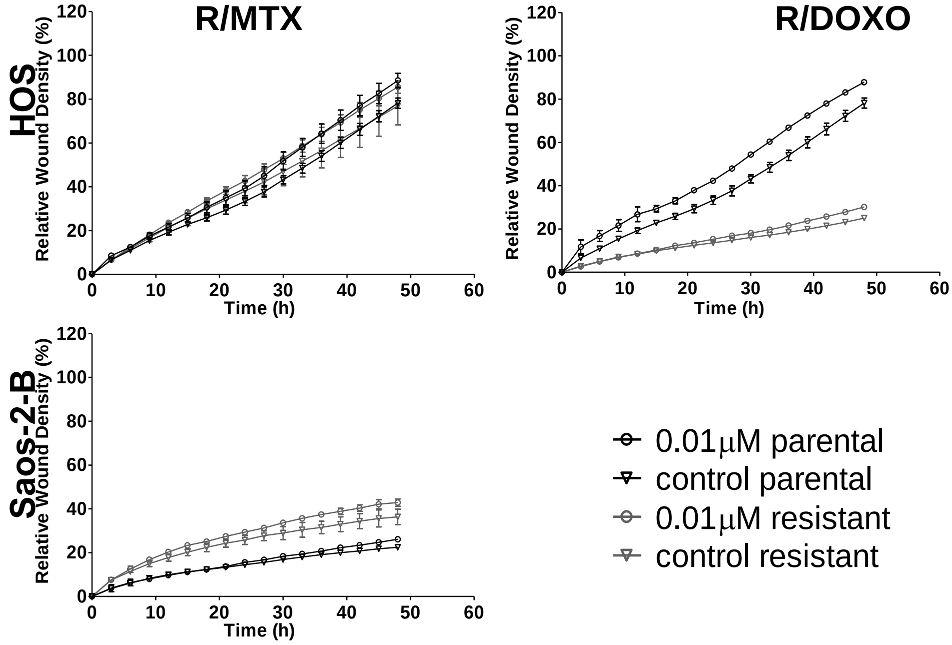

Figure S1: ***In-vitro* characteristics of HOS and Saos-2-B parental and resistant cell lines to MTX and DOXO.** **A**- Morphology. Cells were cultured in medium for several days, observed under the microscope and a phase-contrast photographs were collected with the IncuCyte system; **B**- Doubling time. Cell lines were cultured in medium for several days and doubling time was assessed using the IncuCyte system; **C** -Migration potential with (0.01µM) and without (control) treatment. MTX resistant cell lines were treated with MTX and the cell line DOXO resistant were treated with doxorubicin (DOXO). Parental cell lines were treated with MTX and Doxorubicin. The same procedure was performed for the others cell lines (143-B-R/MTX, Saos-2-R/MTX, MG-63-R/MTX) and with the others compounds (ETOP, CISP, MAF) showing a similar behavior as HOS-R/MTX.

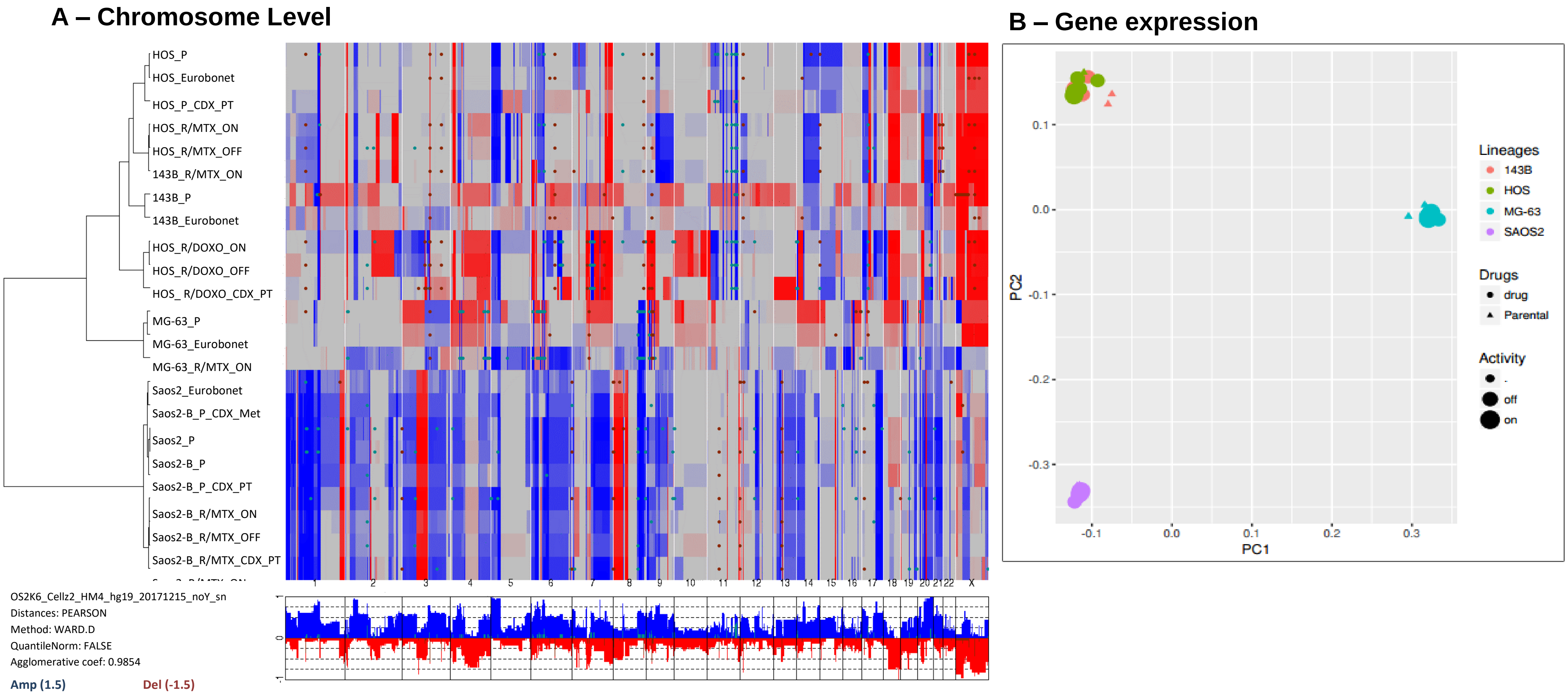

Figure S2: Clustering analysis of the resistant and parental cell lines. A- Hierarchical clustering and heatmap of the copy number abnormalities (CNA) profiles (Pearson distance, Ward construction method). Gains are displayed in blue, losses in red, grey for normality. Amplifications (L2R > 1.5) and deletions (LRR < 1.5) are represented as turquoise and brown dots, respectively. Lower part of the plot represents for the sample population the cumulative frequency of CNA events for gains in blue, and losses in red, along the genome. **B**- First two principal components extracted from principal component analysis (PCA) performed with the gene expression of top 1000 variable genes.

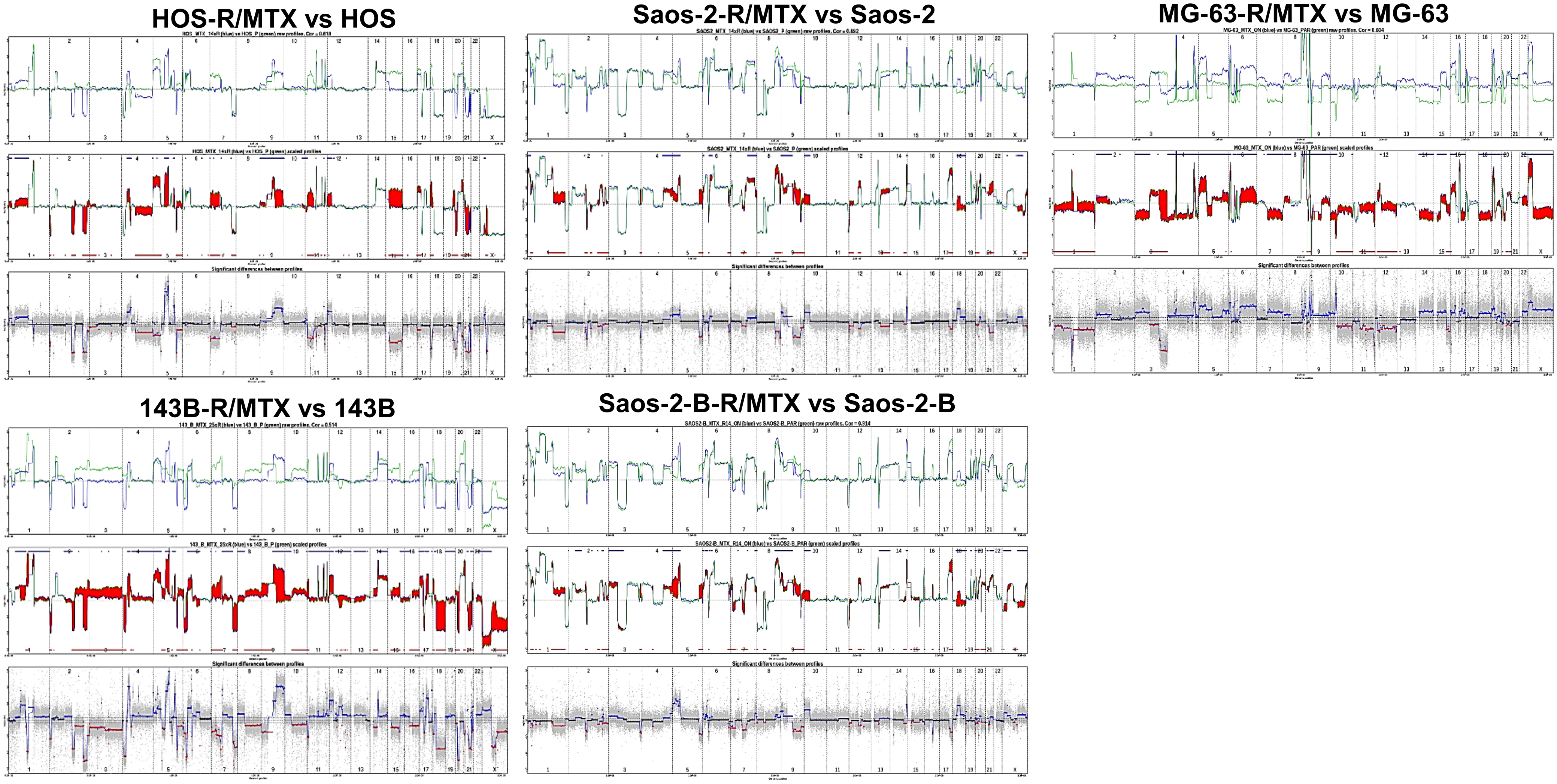

Figure S3: Direct comparison of HOS-R/MTX, 143B-R/MTX, Saos-2-R/MTX, Saos-2-B-R/MTX and MG-63-R/MTX versus their respective parental CNA profiles. Upper panel: Unscaled CNA profiles for MTX-resistant (blue) versus respective parental (green) for HOS, 143B, Saos-2, Saos-2-B and MG-63. Middle panel: Same profiles after dynamics scaling of each parental profile, with significant differences colored in red areas, with corresponding segments positions as blue or red bars. Lower panel: segmentation of the difference profile.

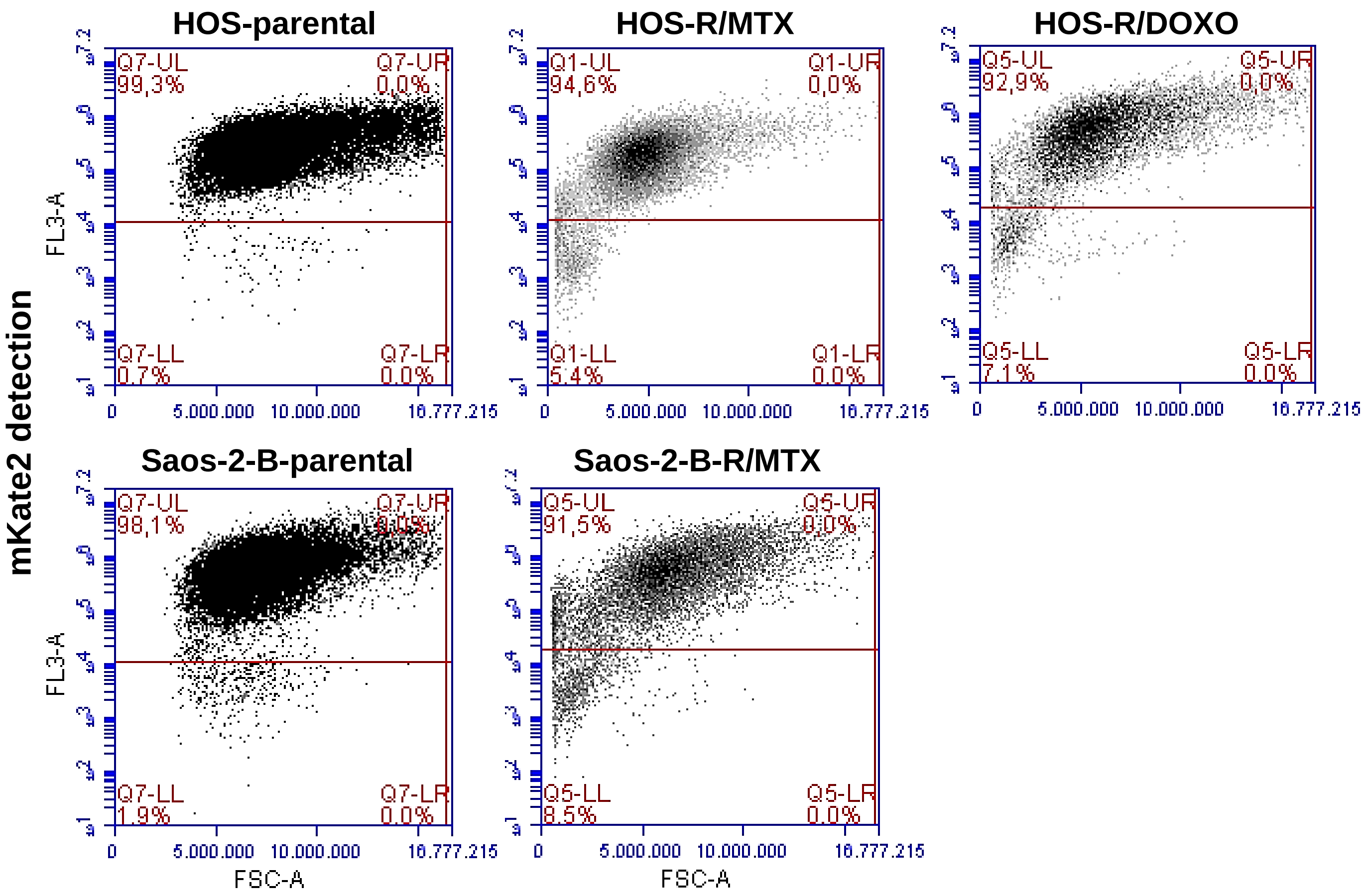

Figure S4: Characterization of luciferase-transfected osteosarcoma cells. Data shown for the two osteosarcoma parental cell lines (HOS-parental and Saos-2-B-parental) and for the respectively methotrexate and doxorubicin resistant cell lines (HOS-R/MTX, HOS-R/DOXO and Saos-2-B-R/MTX) after FACS selection showing a rate of more than 90% of luciferase positive cells.

HOS-Parental-CDX vs Origin Cell line

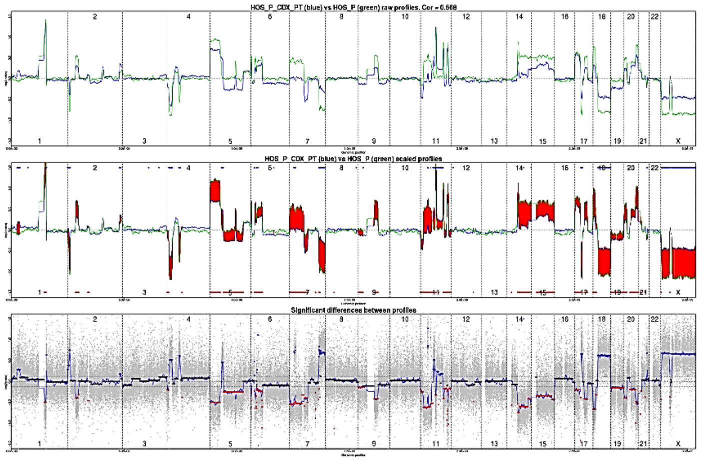

HOS-R/MTX-CDX vs Origin Cell line Drug-ON

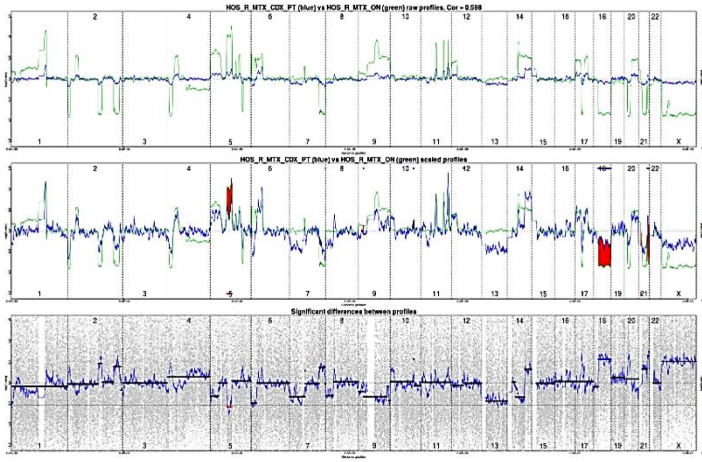

HOS-R/MTX-CDX vs Origin Cell line Drug-ON

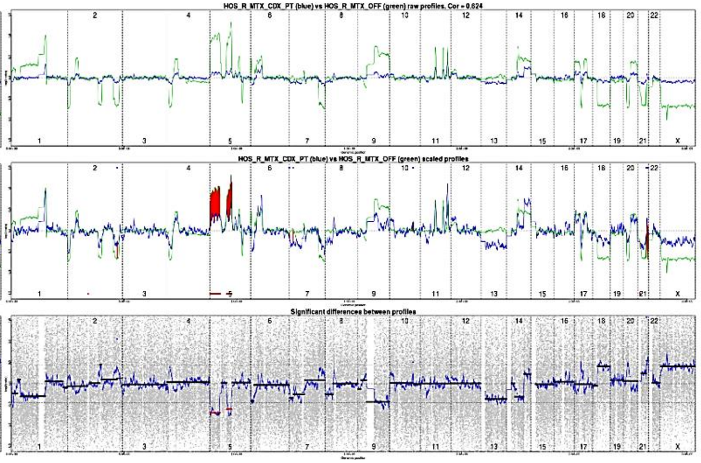

HOS-R/DOXO-CDX vs Origin Cell line Drug-ON

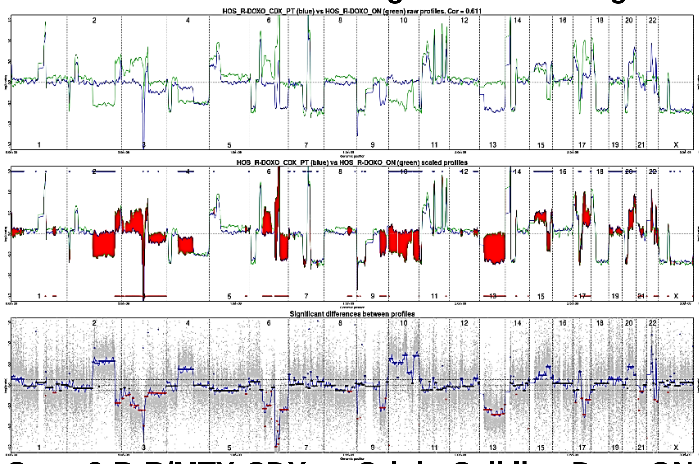

HOS-R/DOXO-CDX vs Origin Cell line Drug-OFF

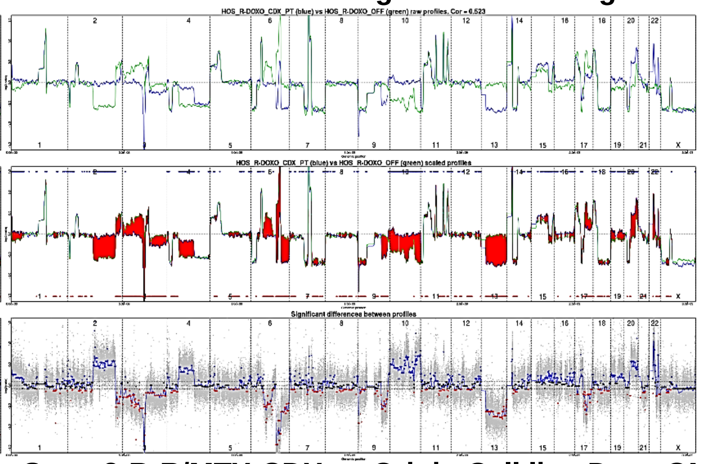

Saos-2-B-Parental-CDX vs Origin Cell line

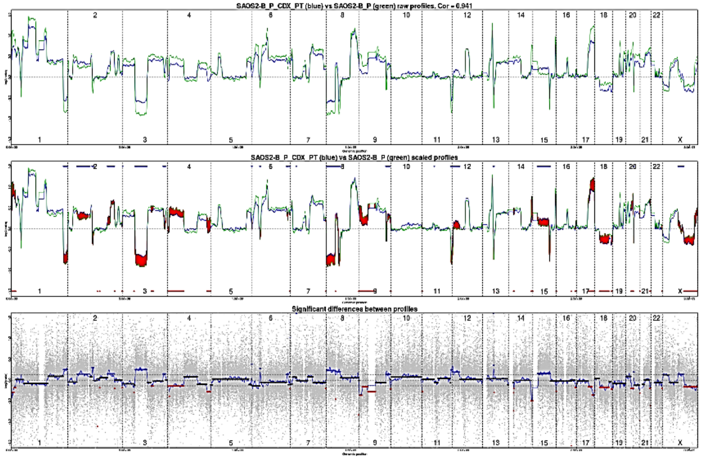

Saos-2-B-R/MTX-CDX vs Origin Cell line Drug-ON

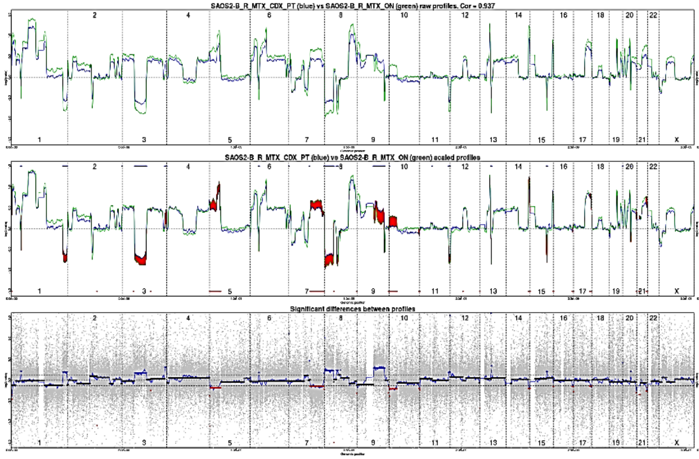

Saos-2-B-R/MTX-CDX vs Origin Cell line Drug-ON

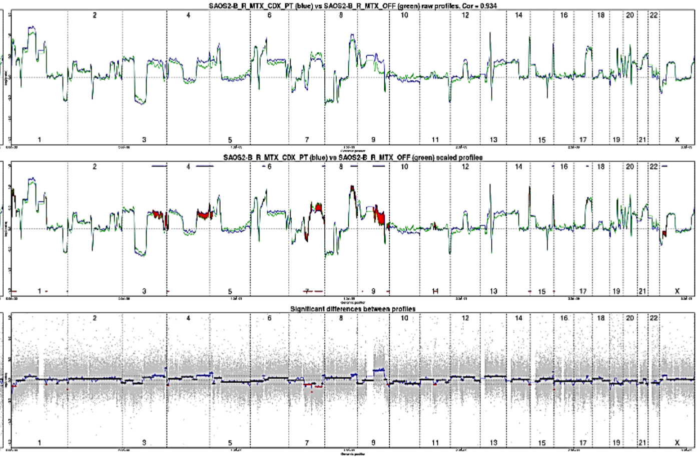

Figure S5: Direct comparison of CDX models versus their respective origin cell line (*in-vitro*) (cell line from which they were issued of) CNA profiles. Upper panel: Unscaled CNA profiles for CDX model (parental or resistant CDX) (blue) versus respective origin cell line (green). Middle panel: Same profiles after dynamics scaling, with significant differences colored in red areas, with corresponding segments positions as blue or red bars. Lower panel: segmentation of the difference profile.

Table S2: Morphological and histological characteristics of all osteosarcoma bioluminescent orthotopic CDX: HOS-parental-CDX, HOS-R/MTX-CDX, HOS-R/DOXO-CDX, Saos-2-B-parental-CDX and Saos-2-B-R/MTX-CDX. BLI- *In-vivo* and *ex-vivo* bioluminescence; CT-Computed Tomography; Histo-Histology; FB-fibroblastic subtype; OB- Osteoblastic subtype; HG- High-Grade osteosarcoma; NA- Not Available; ND- Not done; **+** - Positive detection; **-** - Negative detection; Met-Metastases.

Cell line Luc/mKate2	Resistance	Primary tumor				Metastases					
		Histo	BLI	CT		Lung		Bone		Spleen	
		Sub-type		Calcification	Osteolysis	Histo	BLI	Histo	BLI	Histo	BLI
HOS	Parental	HG FB+OB	++	+	++	++	++	-	-	+	+
		HG FB+OB	++	+	++	++	++	-	+	-	-
		HG FB+OB	++	+	+	-	+	-	-	-	-
		HG FB+OB	++	+	+	-	+	-	-	-	-
		HG FB+OB	++	+	+	-	+	-	-	-	-
	R/MTX	HG FB+OB	+	+	+	-	+	-	-	-	-
		-	+	-	-	-	-	-	-	-	-
		HG FB+OB	+	+	NA	-	+	-	-	-	-
		-	+	NA	NA	-	-	-	-	-	-
		-	+	NA	NA	-	+	-	-	-	-
		HG FB+OB	+	+	+	-	-	-	-	-	-
		-	+	+	+	-	-	-	-	-	-
		+	+	-	+	-	+	-	-	-	-
	R/DOXO	HG FB+OB	+	-	+	-	+	-	-	-	-
		HG FB+OB	+	-	+++	+	+	-	+	-	+
		HG FB+OB	+	-	+	-	+	-	-	-	-
		-	+	-	-	-	-	-	-	-	-
Saos-2-B	Parental	HG OB	+++	++++	+	-	++	-	ND	+	+
		HG FB+OB	+++	++++	+	++++	++++ (Visible)	-	ND	-	+
		HG OB	+++	++++	+	+	++++ (Visible)	-	ND	+	+
		HG OB	+++	++++	+	+++	++++ (Visible)	+	+	-	+
		HG OB	+++	++++	+	+	+++	+	+	-	+
	R/MTX	HG FB+OB	+++	++++	+	+	++	-	-	-	-
		HG OB	+++	+++	+	-	+	-	-	+	+
		HG FB+OB	+++	+++	+	-	+	+	+	+	+
		HG FB+OB	+++	++++	+	+	+	-	-	-	-

Table S3: Characteristics of metastases of all osteosarcoma bioluminescent orthotopic parental and resistant CDX models.

Cell lines	Metastasis number per mice (all organs)	Metastasis maximum Diameter
HOS P	Median 12 (range 2 – 29)	<1mm
HOS R/DOXO	0 - 2	0.1mm
HOS R/MTX	0 - 1<br>(detected by bioluminescence)	Not available<br>(not detected by histology)
Saos-2-B P	Median 15 (range 2 – 32)	0.1 - 6mm
Saos-2-B R/MTX	1 - 5	<1mm

Table S4: Characteristics of Osteosarcoma cell lines. Patients and samples characteristics from where the cell lines were derived and the principal genetic alterations [40–42]. NA-not available

Cell line	Patients and samples characteristics			Principal Genetic alterations				Commercial origin
	Gender	Age	Tumor sample	TP53	RB1	CDKN2A	ATRX	
**HOS**	Female	13	Primary biopsy from distal femur	Mutated (p.Arg156Pro)	Normal	Homozygous deletion	NA	Provided by ITCC
**HOS-143B**	Female	13	HOS virally transfected with Ki-ras oncogene	Mutated (p.Arg156Pro)	Normal	Homozygous deletion	NA	Purchased at ATCC
**Saos-2**	Female	11	Primary biopsy	Del>EX4/EX8	Mutated	Normal	Normal	Provided by ITCC
**Saos-2-B**	Female	11	Saos-2 cells issued from two different culture batches exhibiting slightly different CGH profiles (supplementary-Fig.S2)	-	-	-	-	Provided by ITCC
**MG-63**	Male	14	Primary biopsy	First intron Rearrangements	Normal	Homozygous deletion	Homozygous deletion	Provided by ITCC
**IOR/OS18**	Male	33	Metastatic biopsy	Del>EX3/EX4	Normal	Homozygous deletion	NA	Provided by ITCC

Table S5: Primers used to amplify topoisomerase IIa (TOPO2A), multidrug resistance protein 1 (MDR1/ABCB1) or P-glycoprotein 1 (PgP) and multidrug resistance associated protein 1 (MRP1/ABCC1) cDNAs by quantitative real-time PCR. Glyceraldehyde 3-phosphate dehydrogenase (GAPDH) was used as control.

Gene	Forward primer	Reverse primer
**MDR1/ABCB1**	5′-TGGAGGAAGACATGACCAGG-3	5′-CAAGACCTCTTCAGCTACTGC-3′
**MRP1/ABCC1**	5′-TCTACCTCCTGTGGCTGAATCTG-3	5′-CCGATTGTCTTTGCTCTTCATG-3′
**TOPO2A**	5′-TTGAAGACGCTTCGTTATGGG-3′	5′-CCATCACAACTGGCCCTCTC-3′
**GAPDH**	5′ ATCCCATCACCATCTTCCAG-3'	5′ CCATCACGCCACAGTTTCC-3'
